# Embodied sociotechnical imaginaries: how donor-conceived people imagine identity, family and reprodigital futures beyond regulation

**DOI:** 10.3389/fgwh.2023.1221913

**Published:** 2024-01-11

**Authors:** Giselle Newton, Kerryn Drysdale, Christy E. Newman

**Affiliations:** ^1^ Digital Cultures and Societies, The University of Queensland, Brisbane, QLD, Australia; ^2^ Centre for Social Research in Health, UNSW Sydney, Sydney, Australia

**Keywords:** imaginaries, qualitative, Australia, donor conception, donor-conceived, assisted reproductive technologies, policy futures

## Abstract

Sociological scholarship has begun to explore imaginaries of family and reproduction, yet less work has focused on the emerging social form of the donor family. In this article, we consider the embodied sociotechnical imaginaries of donor-conceived people, exploring their reflections, judgements, hopes, and predictions regarding donor conception. Combining reflexive thematic analysis of free-text survey responses from sperm donor-conceived (*n* = 90) and egg donor-conceived (*n* = 1) and data from semi-structured interviews with sperm donor-conceived people (*n* = 28), conceived in both clinical and non-clinical contexts in Australia, we analyse donor-conceived people's imaginings of family, identity, and the practice of donor conception in the digital age. Our analysis centres the donor-conceived body that imagines, and in doing so, highlights the entanglements of reproductive and digital technologies, and the humans and institutions that drive their uptake. We argue that leveraging the imaginative and political capacities of donor-conceived people is a productive approach that illuminates possible (re)directions of the assisted reproduction industry as well as illustrating potential policy futures.

## Introduction

Individuals engage in a range of imaginative practices in everyday life. Through the process of imagining, we (re)produce ideas about ourselves, our families and relationships, and broader social formations (1). Imaginaries are (re)produced at the level of the individual, yet they are inherently social, located within specific times and places and shaped by sociocultural dynamics including power relations (2, 3). As such, imaginaries of family – who constitutes family and how families can be formed – reflect the sociohistorical contexts from which they emerge. In recent years, rich scholarship from the sociology of family has drawn attention drawing attention to the discursive and ideological aspects of family by foregrounding how “people deploy, even live, *ideas* and *concepts* of what makes a family” (4, p. 866). This scholarship has highlighted how ideas and imaginings “do not cause practice: they *are practices*” in that they are always embodied and affective, and thus materially manifest [(1), pp. 538–539; see also (3)]. Attention to imaginaries of the family, then, “enable insight into the normative expectations and anticipations of ideal family relationships” (5, p. 1424). Yet, we are only beginning to understand how imaginaries reflect the increasing diversification of family formations. The donor family (that is, familial configurations created through the practice of donor conception) is an emerging social form in many high-income countries without established social scripts that work to structure how we think, and therefore enact, families (6, 7). Several important studies have begun considering donor family imaginaries as an alternative way to (re)think family formations that exceed the heterosexual nuclear family (3, 4, 8). For example, Grace and colleagues (8) explored how heterosexual parents, in their imaginaries of sperm donors, both negated the donor's personhood while inscribing the donor's importance through talk of resemblance and heritability. In a similar vein, Nordqvist (4) explored the imaginaries of heterosexual and lesbian parents, and grandparents, in egg, sperm and embryo donor families, elucidating how “genetic normativity” governs family life in a way that genetic relationships are perceived as strong and non-genetic relationships as less durable. Finally, Hudson's (3) study on embodied reproductive imaginaries focused on how donor egg mothers imagined themselves, the donor's motivations and characteristics, and the future (donor-conceived) child. Yet, no research has considered how donor-conceived people – those most affected by the practice of donor conception – might imagine themselves and others in relation to the (evolving) practice of donor conception.

In this article, we consider donor-conceived people's reflections, hopes, judgements, and predictions about donor conception in the digital age. Drawing on data from a national online survey with sperm donor-conceived (*n* = 90) and egg donor-conceived (*n* = 1) and semi-structured interviews (*n* = 28) with sperm donor-conceived people, we explore diverse imaginings of family, identity and the practice of donor conception. In interpreting accounts of donor conception through the lens of embodied sociotechnical imaginaries, our conceptualisation illuminates the entanglement of reproductive and digital technologies, and the humans and institutions that use, govern or resist them. By centring the donor-conceived body that imagines, we consider the political imperative to engage with the ways donor-conceived people make sense of the past and share their hopes for the future. More broadly, our focus may offer more nuanced understandings of modern families, insights into the emergence of new collective identities, as well as knowledge about the evolving landscape of reproductive technologies and associated policy and regulation.

### (Non-) regulation in donor conception: moving beyond binaries?

Practices of donor insemination have existed since the early 19th century, at least, but until the mid-1980s, no countries regulated the practice (9, 10). During this period of unregulated clinical donor conception, donors were promised anonymity by doctors, and parents were encouraged to maintain secrecy over the details of the conception (11). From the mid-1980s, donor conception regulation was introduced in some jurisdictions, such as Sweden and Victoria, Australia, although legislative regimes were devised to govern clinic-based practices only (9). Attitudes to secrecy and anonymity have gradually shifted over the years and today the importance of donor-conceived people's right to know their donor conceived status and access information about the donor's identity is recognised in some state, national and international policy (see 12–14). Accordingly, in recent years, some jurisdictions have introduced retrospective legislation to facilitate equitable access to information for donor-conceived people conceived in clinical contexts (15).

Outside the clinic, since at least the 1970s, self- or home-insemination has been practiced by lesbian and single women with sperm from a person known to them (16–19). In recent years, social media has facilitated new non-clinical practices, including avenues to finding “online sperm donors” or promoting one's availability and willingness to donate sperm via digital means (18, 20, 21). As such, the scale of non-clinical donation today is much greater than the pre-digital age, oftentimes without the same expectations and obligations between parties (18, 22). Regulators have emphasised the medical, legal, safety, and relational risks of these forms of non-clinical insemination, such as transmission of infections, undesirable legal parentage outcomes, coercive behaviours and large sibling cohorts (23–25). It has been suggested that non-clinical approaches to finding a donor and conducting insemination have democratised donor conception (22), and therefore to some extent threaten the business model of the assisted reproduction industry because recipient parents are able to conceive within the home, for free. In summary, non-clinical conception is difficult to regulate (and monitor) because it is conducted outside the fertility sector, although in some jurisdictions laws dictate maximum offspring numbers (18). In contrast, regulation of donor conception within the clinical context is possible but relatively recent and limited in scope. This means that conflation in terminology of “regulated” with “clinical” obscures the lived experience of those conceived within unregulated clinical contexts, representing an injustice to those generations (see also 26). Similarly, the “known” and “unknown” dichotomy is largely obsolete in an era of direct-to-consumer DNA testing and social media, in which donor-conceived people are able to circumvent regulation to identify donors and donor siblings (27).

### Beyond regulation: capturing donor-conceived people's agentive digital practices

Over the last decade a small yet rich body of work has begun to explore the experiences of donor-conceived people (28, 29), with research beginning to capture how this population understand, contest, strengthen, and sidestep regulation (27, 30, 31). Studies conducted with donor-conceived children and adolescents have found that where disclosure of donor conception status occurred during early childhood, youths felt comfortable, positive or neutral about their conception and were curious about their donor or had already established a positive relationship with them (32–34). Similarly, research with donor-conceived adults has revealed that many desire information about and contact with their donor and donor siblings (10, 35–37). Yet, due to the (lack of) regulation in place during conception, or regulation that restricts provision of information until adulthood, many donor-conceived people are not able to access information about their donor or donor siblings during childhood and adolescence. Important scholarship by Klotz (30) has pointed to how donor-conceived people resist “authoritative regimes of ‘kinship knowledge-management’” through digital and genetic technologies (p. 48). Indeed, since 2000, donor-conceived peers have been interacting online, initially via Yahoo groups, which were superseded in the following decade by closed Facebook groups (26, 38, 39). Such social media platforms have not only been key to community formation but also activist organising for legislative reform and exchange of information about direct-to-consumer DNA testing (27, 30, 40, 41). DNA testing offers many donor-conceived people more (timely) information than government donor registries, given that testing can be conducted in a short timeframe, at any age and offer a broader set of “matches” beyond the donor and donor-conceived person (22, 27).

Access to digital technologies for initiating and sustaining donor family relationships raises questions about the role of regulation in a digital age. There is an urgent need for empirical research to inform more rigorously conceptualised understandings of how donor-conceived people imagine their relationships with others (e.g., family members, peers) and with evolving technologies (e.g., reproductive, digital, genetic), vis-à-vis shifting regulatory and policy environments. While researchers have noted that donor-conceived people born in the 1970s and 1980s represent some of the strongest proponents in movements to end secrecy and anonymity, and have long been speaking publicly to educate the general public, policymakers and assisted reproduction providers (9, 29, 42, 43), little research has theorised how donor-conceived people imagine their roles in creating social change in practices of donor conception.

### Conceptualising embodied sociotechnical imaginaries

A key concept for understanding relationships and social change is the imaginary. For instance, Smart (2) emphasised the ways in which imaginaries are:

not limited to individual or personal imaginings but also connects with the social and cultural level […] our personal musings, desires, thoughts and emotions about and around relationships are not entirely individual because they are formed in social and historical contexts (p. 49).

In this article, we draw on conceptualisations of embodied imaginaries as theorised by Dawney (1) and Hudson (3). Specifically, we find utility in an analytical attention to “*the body that imagines*” in recognising that imagining is a situated practice, shaped by power relations and political capacities (1, p. 539). Such an approach illuminates how a subject position and associated lived experience, such as that of a donor-conceived person, shapes particular social imaginaries that are both backward- and forward facing: the *embodied imaginer* ponders the past, present and future. We draw this work into dialogue with scholarship on sociotechnical imaginaries, a useful framing when considering assisted reproduction given the entanglement between technical and digital processes, and the humans and institutions that drive their uptake (44). Sociotechnical imaginaries, according to Jasanoff and Kim (44) are “collectively held, institutionally stabilized, and publicly performed visions of desirable futures, animated by shared understandings of forms of social life and social order attainable through, and supportive of, advances in science and technology” (p. 4). Applying such an approach to the context of donor conception – as a reproductive technology – draws attention to how individuals envisage the material, social and moral terrains that are shaped by (and in turn shape) the technology. Specifically, our analysis that follows is focused on donor-conceived people's deliberations, hopes, uncertainties, judgements, expectations and predictions as they situate themselves as products of reproductive technologies and position themselves temporally and socially in relation to their peers, their family, and other actors within donor conception.

## Methods and materials

This article forms part of a multi-method study focused on Australian donor-conceived people's experience, perspectives and support needs. The project was led by GN, a donor-conceived woman conceived in a clinical context to heterosexual parents in Australia. Prior to the commencement of the study, GN had interacted with other donor-conceived adults online. Our approach to recruitment, which sought a diverse sample of donor-conceived people from distinct family formations, was also shaped by KD and CN's knowledge and experiences of queer families and sustained relationships with LGBTQ+ organisations (see 45). Ethics approval for the study was provided by UNSW Sydney Human Research Ethics Committee (HC190998). Our approach to recruitment was to circulate information about the study across and through a range of existing contexts and channels to recruit donor-conceived participants with the broadest possible range of experiences. For example, participants could learn about the study through media articles, information from partner organisations (Victorian Assisted Reproductive Treatment Authority (VARTA), Victorian Adoption Network for Information and Self Help (VANISH), ACON (AIDS Council of NSW), Thorne Harbour Health Victoria, Intersex Human Rights Australia and Rainbow Families) and word of mouth referrals, advertisements in public and private Facebook groups for donor-conceived people (such as Australian Donor Conceived People Network, and We Are Donor Conceived, as well as a donor conception meme group), and broader donor conception groups (such as Egg donors Australia and NZ, Donor Conceived Best Practices and Connections), Facebook advertisements, and other social media. Previous research has highlighted that online and social media recruitment, including via Facebook, represents an effective methodology for accessing “hard-to-reach” or minority populations, including donor-conceived people (46–48). Recruitment via Facebook, for instance, allows participants to view the researcher's profile and participation history which may improve response rate and increase levels of trust (47).

This article combines data from two methods: a national online survey (*N* = 91) and semi-structured interviews (*N* = 28) which formed two distinct but concurrent arms of a study conducted in 2020. All eligible participants had the option to complete the survey, but a smaller number of interviews were conducted. Data from the survey and interviews were not linked, so it was not known how many participants took part in both. Written informed consent to participate and to publish any potentially identifiable data was obtained at the start of both forms of data collection. The survey sought to describe broad patterns of experience among Australian donor-conceived adults and included questions related to: demographics and donor conception history, support and services, and digital technology use and advocacy. Donor-conceived people who were Australian citizens or permanent residents and over 16 years old were eligible to participate in the survey. Hosted on Qualtrics, the survey required approximately 30 min to complete. At the conclusion of the survey, respondents were invited to register their interest to participate in a semi-structured interview if they were over 18 years old, an Australian citizen or permanent resident and members of one or more Facebook groups for donor-conceived people. But as these arms were independent, it is possible that participants did not complete the survey prior to participating in an interview. Survey respondents learnt about the study from one or more of the following avenues: a family member or friend (*n* = 16), an organisation or service (*n* = 3), an email or message (*n* = 6), a Facebook ad (*n* = 9), a post in a Facebook group (*n* = 54), a post on other social media sites (*n* = 5), a google search leading to the study website (*n* = 3), a media article (*n* = 1).

The semi-structured interviews were exploratory and sought to capture donor-conceived adults' rich, subjective accounts, with a component of the interview focused on digital technologies. At the start of all interviews GN detailed her “insider” position as a donor-conceived person (see 31). All interviews were audio recorded and then transcribed verbatim and de-identified.

Data from the survey and interview methods were mixed during the analysis stage, following Mason's (49) approach in which insights derived from different methods are integrated, consolidated and meshed “to produce a fuller or more valid or more robust picture” (p. 20). The qualitative data – including free-text survey responses and semi-structured interview transcripts – were analysed together, informed by the interpretive traditions within sociology and Braun and Clarke's (50) approach to reflexive thematic analysis. Attention was paid to shared patterns of meaning as well as atypical cases and contradictions within the data. Below, we first provide information about the sample followed by three thematic domains we derived and developed through our analysis: “imagining a donor-conceived (collective) identity”, “imagining the donor family”, and “imagining the practice of donor conception”. Excerpts included in this article are labelled with a pseudonym, family structure, context of conception, and the research method the data is drawn from.

### Study sample

The comparative demographics and conception contexts of the online survey and semi-structured interview participants are displayed in Table 1. The survey sample consisted of 91 respondents: 75 were women, 14 were men and 2 were non-binary people. A total of 13 respondents were conceived in the 1970s (14.29%), 46 in the 1980s (50.55%), 22 in the 1990s (24.18%), and 10 in the 2000s (10.99%). Almost all respondents were conceived with donor sperm (98.9%). The majority of participants were conceived in heterosexual family structures (79.1%), and most were conceived within a clinical context (84.6%). Of the 28 semi-structured interview participants, all participants were sperm donor-conceived, the majority were women (82.1%), from heterosexual parent families (89.3%) and were conceived in a clinical context (96.4%). Like the survey sample, the majority of participants were conceived in the 1980s (67.9%), and 1990s (17.9%), and the remaining participants were conceived in the 1970s (7.1%) or 2000s (7.1%).

**Table 1:** Study sample.

Online survey		Semi-structured interviews	
Gender		Gender	
Women	75 (82.4%)	Women	23 (82.1%)
Men	14 (15.4%)	Men	4 (14.3%)
Non-binary	2 (2.2%)	Non-binary	1 (3.6%)
Family type at time of conception		Family type at time of conception	
Heterosexual parents	72 (79.1%)	Heterosexual parents	25 (89.3%)
Lesbian mothers	8 (8.8%)	Lesbian mothers	1 (3.6%)
Single/solo mother	11 (12.1%)	Single/solo mother	2 (7.1%)
Conception type		Conception type	
Sperm donor-conceived	90 (98.9%)	Sperm donor-conceived	28 (100%)
Egg donor-conceived	1 (1.1%)	Egg donor-conceived	0
Context of conception		Context of conception	
Clinical context	77 (84.6%)	Clinical context	27 (96.4%)
Community context	12 (13.2%)	Community context	1 (3.6%)
Unsure	2 (2.2%)	Unsure	0
Decade of conception		Decade of conception	
1970s	13 (14.3%)	1970s	2 (7.1%)
1980s	46 (50.5%)	1980s	19 (67.9%)
1990s	22 (24.2%)	1990s	5 (17.9%)
2000s	10 (11.0%)	2000s	2 (7.1%)

## Results

### Imagining a donor-conceived (collective) identity: from me to us

The first theme developed through our qualitative analysis encompasses how donor-conceived people imagined their individual and collective identity. New sites of genetic knowledge and emerging reproductive technologies have been found to shape contemporary identity practices, positionings and groupings (27, 51). Our findings revealed how participants attempted to individually and collectively make sense of what being donor-conceived meant to them and their families.

A number of participants conceived in same-sex mother-parented families with a non-clinical context positioned donor conception as a method and indicated that donor conception did not figure in the way they understood themselves:

*For me, being donor-conceived isn't a big part of my identity, and I don't feel that it has shaped my experience in a way that makes me want to reach out and relate to others with similar situations* (Morgan, same-sex mothers, non-clinical conception, always known, survey response)

Other participants, such as Bailey who was conceived in a non-clinical context by heterosexual parents, explicitly articulated that they imagined their situation as different from other donor-conceived people:

*I feel that my situation is different to people who do not know the donor(s) or who were adopted* (Bailey, heterosexual parents, non-clinical conception, survey response)

The above excerpts reflect how participants resisted (to differing extents) being defined as a donor-conceived person within the context of research focused on Australian donor-conceived people's experience, perspectives and support needs. Bailey, for example, contrasted their “situation” of growing up with a known donor, with the experiences of individuals who identify as donor-conceived, who do not know the donor and thus have more in common with adoptees who similarly may lack information about their parentage. Indeed, many participants gestured to how secrecy and withholding information could contribute to the extent to which someone identified as donor-conceived. Other participants described how mystery figured in their (new) identity:

*It's just added a whole element of like mystery to my life, you know […] but, for the most part, it's something I just kind of like put to the back of my mind and don't think about too much.* (Olivia, heterosexual parents*,* clinical conception, interview)

Olivia highlighted the enigma associated with her donor-conceived identity (see also 52) and although she sought not to focus on or deal with this aspect of her life, it was always in the back of her mind, and to some extent haunted her (see also 26).

A number of participants, both non-clinically and clinically conceived, described being told they were “wanted” and how this formed part of their conception story and identity. Henry, for instance, reflected on the “special circumstances” of his conception:

*We are created under special circumstances and, you know, were all a little bit lost trying to find, you know, trying to find ourselves with each other.* (Henry, heterosexual parents*,* clinical conception, interview)

Here, Henry imagines the donor-conceived “we” as a group united by a shared narrative of an uncommon conception. Importantly, Henry highlights a path to discover or co-create an identity, a process of becoming made possible through access to, and use of, digital technologies.

Others, like Ruth, pushed back against discourses of being “special”:

*Parents like to think that it means that you're so wanted and so special. But it just to me means that you're a commodity and you are here to serve others and make others happy, and spend your life searching for answers. All humans have intrinsic value, whether they were accidentally conceived on a drunken night out or purposefully conceived in a clinic.* (Ruth, heterosexual parents*,* clinical conception, interview)

Ruth critiqued the narrative about how effort and planning in donor conception made her more unique or valuable than any other person. Instead, Ruth pointed to how the donor-conceived child is a “commodity” or, in Ahmed's phrasing, a “happy object”, for their parent/s [(53), p. 27; see also (54)]. While the child is the solution to the parent/s' happiness, participants often felt that ensuring that biological family members could have a meaningful role in the donor-conceived person's life was overlooked by parents. Therefore, the idea of searching for an (collective) identity and belonging was central in many participants' accounts.

Some participants, especially the eldest among the cohort of participants, recalled what it was like to be donor-conceived before the internet. Fay reflected on the years in which she had no contact with other donor-conceived people which made her feel “lonely” in her donor-conceived identity:

*For a long time, it meant to me that I was quite unique but not in a good way, and felt quite lonely in my identity and not being able to identify with everybody, [or] anybody particularly who had been in similar circumstances.* (Fay, heterosexual parents*,* clinical conception, interview)

In these comments, as well as others later in the interview, Fay painted a before and after picture of an era in which she was not connected to other donor-conceived people. She positioned this experience in contrast with a social media age in which connectivity between donor-conceived people was possible:

*It was just surreal to be able to, you know, read other peoples' stories and go, “Oh my God, that's amazing! That's what I feel like!” And, you know, just all of a sudden having this massive connection to all these people and this support network. Yeah. It just, it was mind-blowing from going to not knowing anybody else to then going and having, you know, at your fingertips, all this information, and then these people who'd experienced the same thing* (Fay, heterosexual parents*,* clinical conception, interview)

Here, Fay describes the feelings of awe and solidarity between donor-conceived peers, made possible through the formation of closed Facebook groups for donor-conceived adults. Fay positions herself as part of a collective of people who “experienced the same thing”, who could then exchange social support and together ascribe meaning to their donor-conceived identity. As such, digital platforms figure in imaginaries of becoming for the donor-conceived collective, a topic also mentioned by Rachel:

*I joined a Facebook group for Donor Conceived adults a few years ago. The members of this group encouraged me to undertake DNA testing (which I previously knew nothing about). They even offered to pay for a testing kit for me. I undertook the Ancestry DNA test and I finally felt as though my identity had been cemented and felt very proud.* (Rachel, heterosexual parents*,* clinical conception, survey response)

Here, DNA testing figures in donor-conceived imaginaries of identity in two ways: as a set of practices that donor-conceived people collectively *do,* and as a tool for consolidating and strengthening individual identity. This figuration takes place, particularly, in relation to positioning within familial networks, as we now go on to explore.

### Imagining the donor family: identifiability and connectedness

The second theme derived from our analysis relates to donor-conceived people's imaginings of donor families over time. Perspectives on who constituted parents and the role of the donor were discussed by all participants, according with previous research that has shown that donor-conceived people's attitudes and experiences differ according to the family structure the person grows up in (10, 36, 55). Therefore, it was somewhat unsurprising that participants gestured to how their parents' sexuality and preferences for (alternative) family forms contributed to their imaginings of the donor family. This was especially the case for participants who were conceived in non-clinical contexts – many of whom were raised knowing that they were donor-conceived in LGBTQ+ and/or solo parent families. For example, Ash had always known that they were donor-conceived:

*My mother is a lesbian and wanted to be a mother so asked a friend if they had any friends that would donate sperm. It has never been hidden from me and I have always had the opportunity to find out about my father but have never felt the need.* (Ash, same-sex mothers, non-clinical conception, survey response)

The embodied imaginary of donor families above is characterised by openness and underpinned by the belief that family included other LGBTQ+ families, friends, and community. Put differently, some participants highlighted the value of chosen families over bio-genetic relationships (56, 57).

However, not everyone conceived outside a clinical context understood that they were donor-conceived or grew up alongside members of their donor family. For example, Bobbie described her feelings of shock upon learning she was donor-conceived at eight years old:

*Realising two men or two women couldn't make a baby was a huge revelation for me, and I had no-one to talk to except my mothers. And that's when I realised my mothers were keeping a secret from me, you know, bigger than Santa Claus.* (Bobbie, same-sex mothers, non-clinical conception, interview)

Here, Bobbie's family consisted of her two mothers until she learnt that her mothers were not able to conceive alone. In this sense, the pre-established embodied imaginary of family was disrupted through this lesson in biology. More broadly, Bobbie's account challenges the widely held assumption that it is obvious to children born in single/solo and same-sex parent families that their conception story is non-normative. This account also suggests that disclosure in childhood (e.g., age eight), can be met with negative emotions such as shock (see also 55).

Bobbie went on to explain how, in order for her existence not to threaten the donor's nuclear family dynamic, an arrangement in which contact was prevented was forged:

*They knew who he was and because their agreement with his wife and him was that I would never know he was meant to be anonymous my whole life to protect their daughters […]it was a huge secret. There's always a level of trust there that I think is severed. You know, it's a pretty big thing to keep from a kid.* (Bobbie, same-sex mothers, non-clinical conception, interview)

Bobbie describes her negative attitudes towards the conception pact and how such an arrangement impacted her sense of trust in her parents (see also 27). More broadly, Bobbie's experience aligned more closely with that of many participants who were conceived in a clinical context in which doctors promised donors anonymity and encouraged parents to keep the information about the child's conception a secret.

Many clinically conceived participants also described a longing to have identifying information about their donor, often initially couched in a need to better understand their health:

*I'd like to know a little bit more about my medical history. Just sort of answers to why I am like how I am, and my personality, how much of it comes from my mum's side, how much does come from my biological father's side.* (Rob, heterosexual parents*,* clinical conception, interview)

Here, Rob positions information about his donor as critical to understanding himself. We see how from the donor-conceived person's perspective, the donor has a presence in the donor family, albeit a nameless, faceless presence (26).

Another participant, Shane, described identifying and meeting their donor and donor sibling in their thirties using information from the donor register, DNA testing, and a Facebook group. Initial digital interactions “through Facebook, Messenger, Skype; all that kind of stuff” eventually led to an in-person meeting:

*It was like meeting someone you've known your whole life for the first time. You recognise yourself in the other person and it's a rush. All the nights laying in bed, wondering, “Who are these people? Will I ever meet them?” […] it just felt normal except I had no idea who these people were. But I knew exactly who they were.* (Shane, heterosexual parents*,* clinical conception, interview)

In their account, Shane underscored the feelings of peace that surfaced as the donor transformed from abstract to material. What would normally be an unsettling experience – meeting such a significant (and formative) figure for the first time – instead was filled with familiarity, predictability and understanding.

In contrast to those who forged donor family relationships later in life through digital means, several participants conceived in a non-clinical context described how they had formed relationships with their siblings and/or donor from a young age:

*I have had contact with four of my siblings all of my life (all conceived through community/friends)* (Max, single mother, non-clinical conception, survey response)

In the excerpt above, Max emphasises the closeness between him and his siblings, in that they belonged to the same “community/friends”. As such, the imaginary of community was more prominent than the imaginary of the donor family for some respondents.

Interestingly, some of the donor-conceived participants conceived via clinical routes viewed non-clinical donor conception as a more positive method in ensuring the donor-conceived person could have a relationship with their biological family from a young age. For example, Monica described learning about a younger peer's experience of non-clinical conception on a Facebook group for donor-conceived people:

*I was like, “Wow! Like if only my parents were like open to that idea that they could have asked somebody that they knew.” And then I grew up knowing that he's like a special uncle or something.* (Monica, heterosexual parents*,* clinical conception, interview)

Likewise, Athena highlighted the challenges of initiating relationships with biogenetic relatives at age 37:

*I get a bit, I don't know that ‘resentful’ is quite the word but maybe envious of kids who grow up connecting younger or with known donors who have parents to navigate this and set relationships so that, by the time they're older, the relationships are established. And I'm 37, and my mum can't do that for me. And I have to do it myself* (Athena, heterosexual parents*,* clinical conception, interview)

Here, Monica and Athena recognise the challenges of initiating familial relationships as an adult and the advantages of selecting a donor who has an existing relationship with the family who could have a “special uncle” role. These excerpts exemplify the embodied sociotechnical imaginaries of donor-conceived people, where through exposure to distinct models for doing donor families via peer Facebook groups, individuals were able to compare and contrast ways of doing donor conception. The “what ifs” and “what could have beens” illustrate a clear path forward, where all members of the donor family are always connected or permanently within reach.

### Imagining the practice of donor conception: the temporal and moral landscape

The third theme developed through our analysis related to participants' views about the practice of donor conception. Previous scholarship with donor conception studies has considered gamete donors' motivation talk and how donors “do morality” in such interactions (58, 59). Just as donors used moral discourses and positionings, donor-conceived people also reflected on the moralities of the practice of donor conception. Participants both reflected on their own conception, as well as how they had conceived or *would* conceive children themselves. For example, one participant, Lindy, who discovered she was donor-conceived via a direct-to-consumer DNA test in her thirties, described passively participating in a Facebook group for prospective recipient parents searching for donors. Lindy deliberated on the moral plausibility of choosing to conceive via donor conception herself:

*I'm 34 and I'm, at the moment, single, and looking potentially at getting pregnant. So I've just kind of been sitting back and looking at that, and wondering whether that's an avenue I want to go down. And I guess I've got a different perspective being a donor child and wanting to make sure that, if I go down there, that I do it in the right way. […] Definitely, having a known donor. Someone that would probably have some form of communication with my child going forward. And probably someone that could provide or has kids already so my potential child would have siblings and know who they are.* (Lindy, heterosexual parents*,* clinical conception, interview)

Here, Lindy outlines the “right way” to conceive via donor conception: with a donor who agrees to be identifiable and contactable from the start of the child's life and whose (other) children are equally identifiable and contactable. In doing so, she also reveals the “wrong way” to conceive – with secrecy and separation – made clear to her through her lived experience of late disclosure.

In the same interview, Lindy also gestured to how she rationalised the decision to conceive with a donor both to herself and her peers. Lindy later explained how, as a donor-conceived single woman in her mid-30s, she could justify to herself the decision to have a donor-conceived child, yet she feared that if she mentioned the conundrum to her peers in the donor-conceived Facebook group she would face moral judgement:

*I don't know if they'd be so blunt as to say, “You shouldn't do this. Look what we've been through!” I don't know. It's just, I feel like most people in that group have a negative view about donor conception in general. So, I feel like I would possibly get negative comments back* (Lindy, heterosexual parents*,* clinical conception, interview)

Lindy, like other participants, reflected on the negative views held by donor-conceived people toward the practice of donor conception. Specifically, this excerpt illustrates the perceived underlying belief that donor-conceived people should learn from what “we’ve been through”; that is, never repeat the reproductive decisions made by their own parents, which for many donor-conceived people, have contributed to emotional turmoil and hardship associated with (re)establishing identity. Yet, in some cases, and especially for those who would need a donor due to their sexuality or single status, this created moral tensions.

Beyond donor-conceived people's own reproductive choices, it became clear that many donor-conceived participants had sought to shape the conditions for future generations of donor-conceived people. One participant, Sam, described how the representation of queer families had shifted over the years, and how they had actively contributed to change social attitudes:

*The media were generally quite hostile growing up [to queer families] but I've had positive responses as an adult writing opinion pieces about my family.* (Sam, same-sex mothers, non-clinical conception, survey response)

Similarly, Athena, described how she sought to influence prospective recipient parents' reproductive decisions through her participation in a mixed or triad Facebook group, which include gamete donors, (prospective) recipient parents and donor-conceived people:

*There are people who have said to me or to us as a group “I've chosen a known donor situation,” or “a co-parenting situation”, “because of what I’ve learnt here.” And I think, you know, “You’re American: you could have gone fully anonymous. You’ve really made a decision with the best interests of your child” it's a different child but you know what I mean. Like that child's life is totally different to what it would have been had I not spoken to them or had they not been in that group.* (Athena, heterosexual parents*,* clinical conception, interview)

Athena's attitudes, as visible in the above quote, aligned with those of Lindy: that known donation or co-parenting was the best possible donor conception outcome, and therefore the free labour required to encourage prospective parents to act accordingly was worthwhile and meaningful to her (see also 60).

Other participants also felt that parents were investing more time in understanding donor conception and its impacts on the child prior to conceiving:

*I definitely noticed, like over the past like year and a half, parents wanting to conceive are way more aware of the effect it has on donor children. And that's great that that's even being considered because I don't think it was for a really long time.* (Bobbie, same-sex mothers, non-clinical conception, interview)

At the same time, other participants held strong opinions that, even for those conceived in the “right way”, support and services would always be necessary in the context of donor conception:

*Even if we think we're creating people with ART [Assisted Reproductive Technologies]in what we think are the best possible circumstances, recognising that there might still be a need for support. It's gonna be particularly important in the future I think. I feel like there's still a bit of a narrative within ART that, if we do it in the right way, then the people that we create as a result of these practices aren't going to need support, and I just don't think that's true.* (Rosie, heterosexual parents*,* clinical conception, interview)

In this account, Rosie, who had been engaged in community building and advocacy for over a decade, countered broader discourses and predictions in assisted reproduction. Importantly, Rosie could only imagine a future in which donor-conceived people required support and services. Such collective imaginings, anticipation, and associated advocacy efforts by donor-conceived people like Rosie, illuminated possible courses of action.

Across the study, participants acknowledged the progress achieved in donor conception while also foreshadowing the increasing challenges:

*It is a global industry now, you know, in a way that I don't think it was when I was conceived. I think that in some ways the problem's gotten easier to deal with and there's more awareness of donor-conceived people and their need for information. But, in other ways, I think it's become a whole lot more complicated because now we're talking about gametes crossing (international) borders and we're talking about people, going into fertility tourism* (Mabel, heterosexual parents*,* clinical conception, interview)

This excerpt reveals participants' sensitivity to how regulations within a jurisdiction were circumvented through importation/exportation of gametes as well as travel to access assisted reproduction in locations where the practice is unregulated. This suggests that the “problem” of the unregulated landscape and secrecy has now been replaced with the “problem” of international flows of gametes and geographical distance between members of the donor family.

Several participants described feeling disillusioned with a lack of progress in reform on donor conception and detailed how they had experienced burnout from advocacy efforts:

*I just got really exhausted by it and I just felt like the whole thing was insurmountable, and nobody was ever gonna change anything, and it was just such a shit show, and it just felt so unfair. To be honest, every time I've tried to take action on it, I just wanna lay down and go to sleep. Even now talking about it is making me feel emotional just thinking about the … oh, gosh, I didn't expect to get really emotional (wipes eyes) but just thinking about how hard it is to get change and how it's such a fight.* (Dominique, heterosexual parents*,* clinical conception, interview)

The above excerpt, like the one below, reflects the resignation felt among some donor-conceived people. Participants recognised that the extent to which they could influence policy was limited as donor-conceived people, compared to other stakeholders, including the power and influence of the reproductive industry:

*I think there are many injustices for donor conceived people and would like to see those rectified. I don't think any of my efforts (small though they admittedly are) have had any impact. In fact, I think that general attitudes in society and government's approach to donor conception is slipping backwards and many are increasingly willing to accept flawed outcomes for donor conceived people to ensure that the fertility industry thrives.* (Louisa, same-sex mothers, clinical conception, survey response)

Despite the sense of defeat at the “thriving” fertility industry, which was perceived to prioritise profit over positive outcomes for donor-conceived people, participants found solace knowing that the industry was “creating their own enemy”, that the growing population of donor-conceived people would lead to an increased pool of future advocates:

*It might end up being that the clinic might have cut off their nose to spite their face a bit by creating so many of us who then will potentially stand up and say, “Actually, this isn't good the way you’re doing things.” I actually think they might be creating their own enemy. Well, literally.* (Mabel, heterosexual parents*,* clinical conception, interview)

These examples demonstrate the range of imaginative practices donor-conceived people engaged in, and how they positioned themselves temporally and morally. Participants hoped for a more responsible form of donor conception whereby donor-conceived people grew up knowing they were donor-conceived, had access to support and services, and could connect with their biological family members across the life course.

## Discussion

In this article, we have drawn on data from an online survey with sperm donor-conceived (*n* = 90) and egg donor-conceived (*n* = 1) and data from semi-structured interviews (*n* = 28) with Australian sperm donor-conceived people to explore their reflections, judgements, hopes, and predictions via the lens of imaginaries. A conceptualisation of donor-conceived imaginaries contributes to what has been described as a “paradigm-in-the-making” (61). Here, building on and integrating recent scholarship on embodied and sociotechnical imaginaries (1, 3, 44), we centre the donor-conceived imaginers themselves. Drawing on one of the largest samples of non-clinically conceived donor-conceived people, in combination with data from donor-conceived people conceived within a clinical setting, our findings illuminate how donor-conceived people imagine their identities and families. Consistent with previous studies on donor-conceived adults' experiences, only those who were aware they were donor-conceived could be recruited, many participants were recruited via online channels (which may shape their experiences and perspectives of donor conception), the majority of participants were sperm donor-conceived and were women (see 11, 36, 40, 46, 55). Previous donor conception research has noted that women may be more likely to participate in research (36) and advised that caution should be taken in extrapolating findings about sperm-conceived people to other groups such as those conceived using third-party oocytes, embryo or via surrogacy (46). Our findings highlighted that for some (sperm) donor-conceived participants, including a number of those non-clinically conceived, donor-conceived was not a label they identified strongly with, and instead, they viewed it simply as a method of family making and/or as a common reproductive practice within their broader community. Other donor-conceived participants made sense of their identity in closed Facebook groups, where they asked questions, sought information, and found resonances in overlapping experiences. Indeed, shared affects and intimacy between peers – from longing, overwhelm, solidarity, anger, burnout to hope – were made possible through digital infrastructures that brought donor-conceived individuals together in communities. Our findings also revealed how donor-conceived participants exploited DNA platforms, which sort and order users by centimorgans, to identify and activate familial networks. In this way, our analysis highlights how digital platforms figure in many donor-conceived people's sociotechnical imaginaries, both as intimate environments and as tools for discovery and answers. In this way, this article contributes to a burgeoning literature (18, 22, 30, 54) which is beginning to capture how digital technologies are (re)configuring donor families. Yet further empirical and conceptual attention is required to illuminate how, as medical technologies behind donor conception become more sophisticated, so do digital technologies. Indeed, processes of *digital mediation* underpin a wide range of donor family interactions both pre- and post-conception, from social media initiated non-clinical conception to interactions on DNA platforms. Andreassen (6) has argued that online media technologies *are* reproductive technologies, in that donor families are entangled in and by media technologies to such a degree that media and reproductive technologies are inextricable. According to Andreassen (6) “mediation contributes to contemporary ‘becoming’ as a family” (p. 4). In order to understand donor families, then, we must consider the merging of fertility and media technologies, and the ways in which donor families *do family* through and in relation to both technologies. Future research could also explore how reprodigital interactions and imaginings can complement and/or undermine regulation. However, understanding the relationship between reprodigital interactions and regulation requires that we both ask new questions about the range of agentive and resisting strategies employed by members of donor families, and also develop new approaches for considering the invisible and ephemeral modes of digital engagement within and between modern families.

The second key contribution of our analysis is located in our focus on embodied aspects of imaginaries. Our findings have foregrounded donor-conceived participants' moral judgements about the landscape of donor conception as a reproductive technology as well as the relational possibilities and consequences of its uptake. Imaginaries “encode not only visions of what is attainable through science and technology but also of how life ought, or ought not, to be lived” (44, p. 4). Our data pointed to many donor-conceived participants' strong sense of *the right way to conceive*, namely with a donor who agreed to be identifiable and contactable from the start of the child's life. These sentiments around early contact align with other parties in donor conception, such as recipient parents and donors, who have been found to value and initiate contact in early childhood via digital platforms (15, 18). Yet beyond moral convictions, our analysis demonstrated the extensive deliberation and advocacy undertaken by some donor-conceived individuals and collectives to achieve social change that is both retrospective (affecting their own rights and relationships) and prospective (shaping the opportunities of future generations). In other words, many donor conceived people “are unwilling to passively accept their fate” (22, p. 3). For example, while some donor-conceived people spoke out in the media about their (queer) families or engaged with possible future recipient parents online, others were engaged in shaping legislative reform processes. In this way, our research builds on recent work by Best and colleagues (62) that has highlighted how donor-conceived people are keen to ensure that donor conception and its impacts are considered not just in terms of childhood but in relation to the full life course. More broadly, we have sought to suggest that a focus on the donor-conceived imaginer – including those conceived within and beyond the clinic – is politically important. As suggested by Daniels (29), attention to donor-conceived voices has “the potential to make us aware of the consequences of [past] policies, practices—both professional and parental—and attitudes—both personal and social” (p. 445). Conceptual attention to imaginaries allows researchers to leverage the political capacities of donor-conceived people, illuminating potential policy futures. We align our findings with those of Hudson (3), who has asserted that a focus on imaginaries is a fruitful avenue:

to open debate regarding the governance and operation of global reproductive bio-economies. It allows us to critique its problematic features and to offer a means by which everyday deliberations about reproductive technologies, such as the ethical use of third party gametes, can be brought into closer dialogue with policy and practice (p. 358).

Donor-conceived people's embodied sociotechnical imaginaries, then, illuminate from the perspective of those with firsthand experience the ways in which donor conception could or *should* be practiced.

Our analysis has also revealed how donor-conceived people's conception histories can shape their own reproductive choices. While previous work has shown that becoming a parent may shape the ways donor-conceived people think about their own conception status (26, 63), this article has highlighted how conception status impacts future parenthood plans. Specifically, our data highlighted how donor-conceived participants who found themselves in a position where a key pathway to parenthood was via donor conception, (e.g., single and LGBTQ+ donor-conceived people) pondered the moral dimensions of if and how to conceive with a donor themselves. For example, some participants felt they would face judgement from their donor-conceived peers if they decided to conceive via donor conception and felt limited in their ability to disclose and discuss their reproductive choices. These insights resonate with work by Mahlstedt and colleagues (43), which found that the majority of donor-conceived participants indicated that they would not conceive using donor conception nor donate gametes themselves and 12.9% believed sperm donation should not be practiced at all. As such, while it is clear that sexuality (and relationship status) shapes pathways to parenthood, and the majority of intending parents seeking to conceive with a donor are single and lesbian mothers (64); [Power et al., (65)], our data illuminates the complex interplay between conception status, sexuality/relationship status and reproductive plans. These findings raise questions and avenues for future work in regards to whether donor-conceived status might shape the ways in which individuals make sense of their own sexuality and/or select pathways to parenthood. More scholarship, then, is required to map the intergenerational impacts of donor conception.

## Data Availability

The datasets presented in this article are not readily available for ethical, epistemological and methodological reasons. Requests to discuss the datasets should be directed to g.newton@uq.edu.au.

## References

[1] Dawney L. Social imaginaries and therapeutic self-work: the ethics of the embodied imagination. Sociol Rev. (2011) 59(3):535–52. 10.1111/j.1467-954X.2011.02015.x

[2] Smart C. Personal Life: New Directions in Sociological Thinking. Cambridge: Polity (2007).

[3] Hudson N. Egg donation imaginaries: embodiment, ethics and future family formation. Sociology. (2020) 54(2):346–62. 10.1177/0038038519868625

[4] Nordqvist P. Genetic thinking and everyday living: on family practices and family imaginaries. Sociol Rev. (2017) 65(4):865–81. 10.1177/0038026117711645

[5] Smith AK Persson A Drysdale K Bryant J Valentine K Wallace J Family imaginaries in the disclosure of a blood-borne virus. Sociol Health Illn. (2021) 43(6):1422–36. 10.1111/1467-9566.13316 34160829

[6] Andreassen R. From the families we choose to the families we find online: media technology and queer family making. Feminist Theory. (2023) 24(1):12–29. 10.1177/14647001211059517

[7] Nordqvist P. Telling reproductive stories: social scripts, relationality and donor conception. Sociology. (2021) 55(4):677–95. 10.1177/0038038520981860

[8] Grace VM Daniels KR Gillett W. The donor, the father, and the imaginary constitution of the family: parents’ constructions in the case of donor insemination. Soc Sci Med. (2008) 66(2):301–14. 10.1016/j.socscimed.2007.08.029 17928116

[9] Allan S. Donor Conception and the Search for Information: From Secrecy and Anonymity to Openness. New York: Taylor & Francis (2017).

[10] Beeson DR Jennings PK Kramer W. Offspring searching for their sperm donors: how family type shapes the process. Hum Reprod. (2011) 26(9):2415–24. 10.1093/humrep/der202 21708794

[11] Frith L Blyth E Crawshaw M van den Akker O. Secrets and disclosure in donor conception. Sociol Health Illn. (2018) 40(1):188–203. 10.1111/1467-9566.12633 29143343

[12] ASMR. Interests, obligations, and rights in gamete donation: a committee opinion. Fertil Steril. (2014) 102(3):675–81. 10.1016/j.fertnstert.2014.06.001 25171952

[13] HFEA. *Disclosure of donor information* (2004).

[14] NHMRC. *NHMRC ethical guidelines for assisted reproductive technology* (2004). Available at: https://webarchive.nla.gov.au/awa/20170818095846/https://www.nhmrc.gov.au/health-ethics/ethical-issues/assisted-reproductive-technology-art.

[15] Kelly F Dempsey DJ. Experiences and motives of Australian single mothers by choice who make early contact with their child’s donor relatives. Med Law Rev. (2016) 24(4):571–90. 10.1093/medlaw/fww038 28137771 PMC5388365

[16] Donovan C. Who needs a father? Negotiating biological fatherhood in British lesbian families using self-insemination. Sexualities. (2000) 3(2):149–64. 10.1177/136346000003002003

[17] Saffron L. Challenging Conceptions: Pregnancy and Parenting Beyond the Traditional Family. London: Weidenfeld & Nicolson (1994).

[18] Volks C Kelly F. The contact expectations of Australian sperm donors who connect with recipients via online platforms. In: Kelly F Dempsey D Byrt A, editors. Donor-Linked Families in the Digital Age. 1st ed. Cambridge: Cambridge University Press (2023). p. 67–84.

[19] Wikler D Wikler NJ. Turkey-baster babies: the demedicalization of artificial insemination. Milbank Q. (1991) 69(1):5–40. 10.2307/3350118 2034184

[20] Bergen N Delacroix C. Bypassing the sperm bank: documenting the experiences of online informal sperm donors. Crit Public Health. (2019) 29(5):584–95. 10.1080/09581596.2018.1492704

[21] Freeman T Jadva V Tranfield E Golombok S. Online sperm donation: a survey of the demographic characteristics, motivations, preferences and experiences of sperm donors on a connection website. Hum Reprod. (2016) 31(9):2082–9. 10.1093/humrep/dew166 27412344 PMC4991659

[22] Kelly F Dempsey D. Donor-conceived families: relatedness and regulation in the digital age. In: Kelly F Dempsey D Byrt A, editors. Donor-Linked Families in the Digital Age. 1st ed. Cambridge: Cambridge University Press (2023). p. 1–12.

[23] FSANZ. *Home insemination using donor sperm—fertility society of Australia and New Zealand* (2023). Available at: https://www.fertilitysociety.com.au/home-insemination-using-donor-sperm/.

[24] Gilman L Nordqvist P. The case for reframing known donation. Hum Fertil. (2023) 0(0):1–8. 10.1080/14647273.2022.2145242 36688598

[25] HFEA. *Home insemination with donor sperm | HFEA* (2023). Available at: https://www.hfea.gov.uk/donation/donors/home-insemination-with-donor-sperm/.

[26] Newton G. On familial haunting: donor-conceived people’s experiences of living with anonymity and absence. In: Dempsey D Kelly F, editors. Donor-Linked Families in the Digital Age: Relatedness and Regulation. Cambridge: Cambridge University Press (2023). p. 154–73.

[27] Newton G Drysdale K Zappavigna M Newman CE. Truth, proof, sleuth: trust in direct-to-consumer DNA testing and other sources of identity information among Australian donor-conceived people. Sociology. (2023) 57(1):36–53. 10.1177/00380385221091184

[28] Blyth E Crawshaw M Frith L Jones C. Donor-conceived people’s views and experiences of their genetic origins: a critical analysis of the research evidence. In: Beier K Brügge C Thorn P Wiesemann C, editors. Assistierte Reproduktion Mit Hilfe Dritter. Berlin, Heidelberg: Springer (2020). p. 361–88.

[29] Daniels K. The perspective of adult donor conceived persons. In: Beier K Brügge C Thorn P Wiesemann C, editors. Assistierte reproduktion mit hilfe dritter. Berlin, Heidelberg: Springer (2020). p. 443–59.

[30] Klotz M. Wayward relations: novel searches of the donor-conceived for genetic kinship. Med Anthropol. (2016) 35(1):45–57. 10.1080/01459740.2015.1012615 25651361

[31] Newton G. Doing reflexivity in research on donor conception: examining moments of bonding and becoming. In: Shaw R, editors. Reproductive Citizenship: Technologies, Rights and Relationships. Singapore: Palgrave Macmillan (2022). p. 279–301.

[32] Blake L Casey P Jadva V Golombok S. “I was quite amazed”: donor conception and parent-child relationships from the child’s perspective. Child Soc. (2014) 28(6):425–37. 10.1111/chso.12014

[33] Scheib JE Riordan M Rubin S. Adolescents with open-identity sperm donors: reports from 12 to 17 year olds. Hum Reprod. (2005) 20(1):239–52. 10.1093/humrep/deh581 15539443

[34] Zadeh S Ilioi EC Jadva V Golombok S. The perspectives of adolescents conceived using surrogacy, egg or sperm donation. Hum Reprod. (2018) 33(6):1099–106. 10.1093/humrep/dey088 29701833 PMC5972639

[35] Dempsey D Kelly F Horsfall B Hammarberg K Bourne K Johnson L. Applications to statutory donor registers in Victoria, Australia: information sought and expectations of contact. Reprod Biomed Soc Online. (2019) 9:28–36. 10.1016/j.rbms.2019.08.002 31956702 PMC6957838

[36] Macmillan CM Allan S Johnstone M Stokes MA. The motivations of donor-conceived adults for seeking information about, and contact with, sperm donors. Reprod Biomed Online. (2021) 43(1):149–58. 10.1016/j.rbmo.2021.04.005 34006483

[37] Nelson MK Hertz R Kramer W. Making sense of donors and donor siblings: a comparison of the perceptions of donor-conceived offspring in lesbian-parent and heterosexual-parent families. In: Claster PN Blair SL, editors. Contemporary Perspectives in Family Research. Vol. 7. Leeds: Emerald Group Publishing Limited (2013). p. 1–42.

[38] Adams D Lorbach C. Accessing donor conception information in Australia: a call for retrospective access. J Law Med. (2012) 19(4):707–21. 22908615

[39] Crawshaw M Daniels K Adams D Bourne K van Hooff JAP Kramer W Emerging models for facilitating contact between people genetically related through donor conception: a preliminary analysis and discussion. Reprod Biomed Soc Online. (2015) 1(2):71–80. 10.1016/j.rbms.2015.10.001 29911188 PMC6001351

[40] Bauer T Meier-Credner A. Circumstances leading to finding out about being donor-conceived and its perceived impact on family relationships: a survey of adults conceived via anonymous donor insemination in Germany. Soc Sci. (2023) 12(3):155. 10.3390/socsci12030155

[41] Newton G Southerton C. Situated talk: a method for a reflexive encounter with #donorconceived on TikTok. Media Int Aust. (2023) 186(1):66–80. 10.1177/1329878X211064646

[42] Macmillan C. A study on the effects of donor conception, secrecy and anonymity, according to donor-conceived adults [Empirical thesis]. Macquarie University (2016). Available at: http://hdl.handle.net/1959.14/1262038.

[43] Mahlstedt PP LaBounty K Kennedy WT. The views of adult offspring of sperm donation: essential feedback for the development of ethical guidelines within the practice of assisted reproductive technology in the United States. Fertil Steril. (2010) 93(7):2236–46. 10.1016/j.fertnstert.2008.12.119 19285663

[44] Jasanoff S Kim S-H. Dreamscapes of Modernity: Sociotechnical Imaginaries and the Fabrication of Power. Chicago: University of Chicago Press (2015).

[45] Newman CE. Queer families: valuing stories of adversity, diversity and belonging. Cult Health Sex. (2019) 21(3):352–9. 10.1080/13691058.2018.1468032 29848226

[46] Adams D. Child welfare paramountcy: the donor conception paradox [Phd thesis]. (2021).

[47] Baltar F Brunet I. Social research 2.0: virtual snowball sampling method using Facebook. Internet Res. (2012) 22(1):57–74. 10.1108/10662241211199960

[48] Guillory J Wiant KF Farrelly M Fiacco L Alam I Hoffman L Recruiting hard-to-reach populations for survey research: using Facebook and Instagram advertisements and in-person intercept in LGBT bars and nightclubs to recruit LGBT young adults. J Med Internet Res. (2018) 20(6):e9461. 10.2196/jmir.9461 PMC6028767 29914861

[49] Mason J. Mixing methods in a qualitatively driven way. Qual Res. (2006) 6(1):9–25. 10.1177/1468794106058866

[50] Braun V Clarke V. Thematic Analysis: A Practical Guide. 1st ed. Thousand Oaks: Sage Publications (2021).

[51] Gibbon S Novas C. Biosocialities, Genetics and the Social Sciences: Making Biologies and Identities. Oxfordshire: Routledge (2008).

[52] Harrigan MM Dieter S Leinwohl J Marrin L. “It’s just who I am … I have brown hair. I have a mysterious father”: an exploration of donor-conceived offspring’s identity construction. J Fam Commun. (2015) 15(1):75–93. 10.1080/15267431.2014.980823

[53] Ahmed S. The Promise of Happiness. Durham: Duke University Press (2010).

[54] Andreassen R. Mediated Kinship: Gender, Race and Sexuality in Donor Families. New York: Routledge (2018).

[55] Jadva V Freeman T Kramer W Golombok S. The experiences of adolescents and adults conceived by sperm donation: comparisons by age of disclosure and family type. Hum Reprod. (2009) 24(8):1909–19. 10.1093/humrep/dep110 19398766

[56] Roseneil S Budgeon S. Cultures of intimacy and care beyond “the family”: personal life and social change in the early 21st century. Curr Sociol. (2004) 52(2):135–59. 10.1177/0011392104041798

[57] Weston K. Families We Choose: Lesbians, Gays, Kinship. New York: Columbia University Press (1997).

[58] Gilman L. The “selfish element”: how sperm and egg donors construct plausibly moral accounts of the decision to donate. Sociology. (2022) 56(2):227–43. 10.1177/00380385211033153

[59] Mohr S. Beyond motivation: on what it means to be a sperm donor in Denmark. Anthropol Med. (2014) 21(2):162–73. 10.1080/13648470.2014.914806 25175292 PMC4200559

[60] Newton G Zappavigna M Drysdale K Newman CE. More than humor: memes as bonding icons for belonging in donor-conceived people. Soc Media Soc. (2022) 8(1):20563051211069055. 10.1177/20563051211069055

[61] Adams S Blokker P Doyle NJ Krummel JW Smith JC. Social imaginaries in debate. Social Imaginaries. (2015) 1(1):15–52. 10.5840/si2015112

[62] Best S Goedeke S Thorpe M. Make our wellbeing a priority: donor-conceived adults call for ongoing support and conversation about their donor conception. Hum Fertil. (2023) 26(2):337–46. 10.1080/14647273.2023.2180439 36905111

[63] Indekeu A Hens K. Part of my story. The meaning and experiences of genes and genetics for sperm donor-conceived offspring. New Genet Soc. (2019) 38(1):18–37. 10.1080/14636778.2018.1549476

[64] Linara-Demakakou E Bodri D Wang J Arian-Schad M Macklon N Ahuja K. Cumulative live birth rates following insemination with donor spermatozoa in single women, same-sex couples and heterosexual patients. Reprod Biomed Online. (2020) 41(6):1007–14. 10.1016/j.rbmo.2020.08.010 33046376

[65] Power J Dempsey D Kelly F Lau M. Use of fertility services in Australian lesbian, bisexual and queer women's pathways to parenthood. Aust N Z J Obstet Gynaecol. (2020) 60(4):610–5. 10.1111/ajo.13175 32424814

